# The Gold-Headed Cane

**Published:** 1874-07

**Authors:** 


THE GOLD-HEADED CANE, for centuries the honored badge of our honorable profession, having served its purpose and now become virtually obsolete in the onward march of knowledge and progress, is destined very soon to be numbered with the things of the past.

The embryo disciple of Esculapius, having passed through the crysalis stage of development and perfected his gaudy wings, with joyous heart and bounding pulse receives from the hand of his alma mater the long-coveted parchment with an afflatus which mounts him at once into the company of full-fledged doctors. Scarcely has he had time to critically inspect the new toy and satisfactorily translate into live English its dead lingo of cabalistic flourishes ere he is reminded that its proper receptacle is the customary jappaned tin box, which, in deference to ethical precedent, is to be forever buried from mortal view, as though infected by some unseen plague, or embodying in its mystical language some deadly secret, which the welfare of the owner and of the public demands should remain forever inviolable. So long as the sentiment of the profession refuses to the public eye this evidence of the qualification of its members for the responsible duties which they are daily called upon to discharge, is it matter of wonder that the world fails to draw a definite line between the qualified and the unqualified practitioner of the art? How is the distinction to be made? What evidence is furnished upon which to base it? May not the quack guard his deadly secret with the same vigilance and care that we do ours? How are the masses to learn to discriminate the quality and reliability of buried credentials?

But, seriously, what harm can result from a qualified practitioner pursuing his vocation under the ægis of his college diploma openly displayed in his business office? We can see none—absolutely none—whilst upon the other hand there is reason to believe that great and substantial *good* might result from the universal observance of such a practice. Teach the public to expect in every physician's office this badge of his vocation, and the absence of it would at once excite inquiry and arouse suspicion. Accustom the public to the daily inspection of such credentials, and the more intelligent would soon learn to properly discriminate them.

Is it an evidence of vanity to display one's credentials in this way? Let the practice become universal and it is at once divested of such a seeming, Is it vanity? What shall we say of the imprint upon any standard medical work, *e.g.:* John Jones, A.M., M.D., F.R.C.P., etc., Member, etc., Physician, etc., Hon., Secretary, etc., and so on?

---

DR. J. P. LOGAN, we learn, has resigned the Professorship of Clinical Medicine in the Atlanta Medical College.

---

WE HAVE RECEIVED the first number (May) of the *Archives of Electrology and Neurology*: a Journal of Electro-Therapeutics and Nervous Diseases. Edited by George M. Beard, A.M., M.D., of New York. The number before us covers 143 pages; it is handsomely gotten up, and promises a career of usefulness and we hope also, of prosperity. It is to be issued semi-annually at $2.50 per year. Supscriptions to F. L. Clacher, No. 107 East Twenty-eighth street, New York.

---

IN THE GALAXY FOR JULY we note a Sonnet by Paul Hayne; Voltaire as a Lover; Poland and the Poles; Life on the Plains; the Voice as a Source of Income; Henri Rochefort, and the Devil and all his Works. "Civil Rights" and social wrongs find no shelter here.

